# A semi-automated workflow solution for multimodal neuroimaging: application to patients with traumatic brain injury

**DOI:** 10.1007/s40708-015-0026-y

**Published:** 2015-12-01

**Authors:** Koon-Pong Wong, Marvin Bergsneider, Thomas C. Glenn, Vladimir Kepe, Jorge R. Barrio, David A. Hovda, Paul M. Vespa, Sung-Cheng Huang

**Affiliations:** 1 Department of Molecular and Medical Pharmacology, David Geffen School of Medicine at UCLA, Los Angeles, CA USA; 2 Department of Neurosurgery, David Geffen School of Medicine at UCLA, Los Angeles, CA USA; 3 Department of Neurology, David Geffen School of Medicine at UCLA, Los Angeles, CA USA; 4 Department of Biomathematics, David Geffen School of Medicine at UCLA, Los Angeles, CA USA

**Keywords:** Traumatic brain injury (TBI), Cerebral blood flow (CBF), Magnetic resonance imaging (MRI), ^15^O-water, ^18^F-FDDNP, Positron emission tomography (PET), Spatial normalization

## Abstract

Traumatic brain injury (TBI) is a major cause of mortality and morbidity, placing a significant financial burden on the healthcare system worldwide. Non-invasive neuroimaging technologies have been playing a pivotal role in the study of TBI, providing important information for surgical planning and patient management. Advances in understanding the basic mechanisms and pathophysiology of the brain following TBI are hindered by a lack of reliable image analysis methods for accurate quantitative assessment of TBI-induced structural and pathophysiological changes seen on anatomical and functional images obtained from multiple imaging modalities. Conventional region-of-interest (ROI) analysis based on manual labeling of brain regions is time-consuming and the results could be inconsistent within and among investigators. In this study, we propose a workflow solution framework that combined the use of non-linear spatial normalization of structural brain images and template-based anatomical labeling to automate the ROI analysis process. The proposed workflow solution is applied to dynamic PET scanning with ^15^O-water (0–10 min) and ^18^F-FDDNP (0–6 min) for measuring cerebral blood flow in patients with TBI.

## Introduction

Traumatic brain injury (TBI) is an important public health and socio-economic problem throughout the world. It is one of the most common causes of death and long-term disability in adolescents, young adults, and the elderly. In the United States, it was estimated that 1.7 million people sustain a TBI annually [1]. Of these people, approximately 81 % were treated in and released from emergency departments, about 16 % were hospitalized and discharged, and approximately 3 % died [1]. However, these numbers underestimate the real prevalence of TBI as they do not account for those people who did not seek for medical care, had non-fatal (mild or moderate) TBI and presented in outpatient settings such as physician’s offices, or those who received medical care from federal, military, or Veterans Affairs hospitals [1].

Non-invasive neuroimaging technologies have been playing a pivotal role in the study of TBI, providing important information for anatomic localization, surgical planning, staging and monitoring the therapeutic responses, and predicting the recovery outcomes that could improve the survival and change management in patients under acute and chronic conditions. Survivors of TBI typically live with varying degrees of physical disability and suffer from significant cognitive deficits (e.g., impaired attention and poor executive function) and psychological health issues (e.g., depression and elevated impulsivity), all of which require long-term or lifelong medical care and support. Advances in understanding the basic mechanisms and pathophysiology of the brain following TBI are somewhat limited due to lack of reliable image analysis methods that allow accurate quantitative assessment of TBI-induced structural and pathophysiological changes seen on anatomical and functional images obtained from multiple imaging modalities. The use of multimodal neuroimaging technologies has the advantages to overcome the limitations of any individual imaging modality and to aggregate clinical characteristics and features obtained from different imaging techniques for knowledge mining and for guiding medical diagnosis and decision making [2]. Some state-of-the-art methods of multimodal imaging and their uses in brain research can be found in the following review articles [3, 4]. While conventional region-of-interest (ROI) analysis enables quantitation of regional changes and serves as the basis for comparing data between individuals both within and across imaging modalities [5], delineation of brain regions through manual ROI drawing is labor-intensive and time-consuming, and is also prone to reproducibility errors [2, 6, 7]. The complexity level of ROI analysis increases tremendously by the complicated nature of TBI that generally involves a combination of focal and diffuse injury mechanisms. Depending on the cause and severity of the brain injury, variability of individual TBI brains is increased, particularly in the presence of focal lesions (e.g., contusion and hemorrhage) and large deformations within the brain (e.g., swelling and enlargement/shrinkage of ventricular space). This adds significant difficulties to conduct group-level analysis (such as statistical parametric mapping, SPM [8]) and poses technical challenges to perform atlas-based anatomical labeling and ROI analysis [9, 10] with minimal or no human intervention. The gist of the problem lies in the use of spatial normalization that integrates brain images obtained from different modalities for the same individuals to establish a one-to-one correspondence mapping between voxels of individual brains and a standard brain template in a common stereotaxic space.

A number of non-linear image registration methods have been proposed for spatial normalization. Some of these methods use a linear combination of trigonometric functions [11] or polynomials [12] as the transformation model. Because of an implicit assumption of small deformations, this class of methods would fail to normalize images with large deformation, resulting in folding, shearing, and tearing of neighboring structures in the original image upon non-linear transformation. More recent research has been geared toward the development of a large deformation framework [13–18] which preserves the continuity of curves and surfaces as well as the boundaries and neighborhoods between structures while allowing a large degree of transformation. Examples of this class of algorithms include Demons [14], LDDMM [18], DARTEL [19] available in the SPM software (http://www.fil.ion.ucl.ac.uk/spm/), FNIRT [20] implemented in the FSL software (http://www.fmrib.ox.ac.uk/fsl/), and symmetric image normalization (SyN) [21, 22] implemented in an open source software package ANTs (Advanced Normalization Tools, [23]), which was built on an Insight Segmentation and Registration ToolKit (ITK) framework (http://www.itk.org/). We used SyN in this study for non-linear brain warping as it can work with different similarity metrics and regularization kernels [23] and has been extensively evaluated with 8 different performance measures using 80 manually labeled MR brain images in a recent large-scale comparative image registration study and was ranked the overall best among 14 non-linear brain warping algorithms being assessed [24].

To address the technical difficulties in analyzing TBI imaging data, we propose and develop a workflow solution framework that combined the use of non-linear brain warping of structural MR images and anatomical labeling to automatically derive the regional cerebral blood flow (CBF) parameters from multi-tracer PET studies. CBF is an important physiological parameter for assessment of brain function in normal and pathological conditions. Brain tissues depend on CBF for delivery of nutrients and for removal of metabolic products. Since Kety and Schmidt developed a theory of inert-gas exchange in 1940s [25], many methods have become available for measuring CBF in human. Currently, PET imaging with ^15^O-water is considered the *gold standard* for non-invasive quantification of CBF [26, 27]. Using a hydrophobic tracer, 2-(1-{6-[(2-[^18^F]fluoroethyl)(methyl)amino]-2-naphthyl}ethylidene)malononitrile (^18^F-FDDNP), the initial uptake (0–6 min) of which is perfusion-limited, it has been shown that regional perfusion can be inferred from the relative-delivery parameter derived by reference-tissue modeling and from the early-summed image, and thus represent surrogate indices of CBF [28]. The use of the proposed workflow solution is illustrated with neuroimaging data obtained from T1-weighted magnetic resonance (MR) imaging and dynamic PET scanning of dual tracers (^15^O-water and ^18^F-FDDNP) on six TBI patients under acute condition.

## Materials and methods

### Subjects and study protocol

The study was approved by the UCLA Institutional Review Board and was conducted under the auspices of the UCLA Brain Injury Research Center. Six patients with acute TBI participated in this study. Written informed consent was obtained from each patient or from their legally authorized representative if the patients were unable to consent for themselves. Patients were admitted to the intensive care unit after initial stabilization or surgical evacuation of an intracranial hematoma and were treated in accordance with published guidelines for the management of severe head injury [29]. Each patient underwent T1-weighted MR imaging and a series of dynamic PET scans. Both MR and PET imaging were performed at the earliest possible time. Delays in PET scanning were commonly attributable to pending informed consent, hemodynamic stability of the patient, surgical procedures, and availability of PET facility, or a combination of one or more aforementioned factors. Table 1 summarizes the demographic data of the patients.

**Table 1:** Demographics of the patients

Subject	Gender	Age (years)	Initial GCS (field)	Initial GCS (ER)	Type of injury	PET (day post-injury)	Glucose at admission (mg/dL)
1	M	26	15	13	MCA	4	124
2	M	34	6	8	MVA	13	122
3	F	54	3	6	MVA	13	152
4	M	35	3	3	MC vs. Auto	10	340
5	M	31	3	3	MVA	8	133
6	M	29	7	9	MCA	5	89

*M* male, *F* female, *GCS* Glasgow coma scale, *ER* emergency room, *MCA* motorcycle accident, *MVA* motor vehicle accident, *MC vs. Auto* motorcycle vs. automobile accident

### Image acquisition

#### MR imaging

A high-resolution structural T1-weighted magnetization-prepared rapid gradient-echo (MPRAGE) MR image was taken for each patient using a 1.5T Siemens Sonata MRI scanner (sagittal plane; repetition time = 1970 ms; echo time = 4.38 ms; inversion time = 1100 ms; field of view: 512 × 512; in-plane voxel size: 0.5 × 0.5 mm^2^; slice thickness = 1 mm; 160 contiguous slices; flip angle = 15°).

#### PET scanning

Each patient underwent a single PET session that consisted of four sequential PET scans (^15^O-CO, ^15^O-water, and ^15^O-O_2_ followed by ^18^F-FDDNP) performed with the ECAT EXACT HR+ scanner (Siemens/CTI) in three-dimensional (3D) acquisition mode. However, only the ^15^O-water and ^18^F-FDDNP PET studies are considered in this paper to illustrate the use of the proposed workflow solution, which is independent of the number of PET tracer studies. Prior to tracer administration, transmission scans were acquired with a set of ^68^Ge rotating rod sources to allow for attenuation correction. Immediately after a bolus injection of ~555 MBq of ^15^O-water through an indwelling venous catheter, dynamic PET scans were acquired for 10 min, with a scanning protocol of 6 × 5 s, 9 × 10, 6 × 30, and 5 × 60 s. Dynamic ^15^O-water PET scans were obtained with concurrent blood sampling via an arterial catheter, where arterial blood samples were taken at 15 time points (0, 5 × 12, 3 × 20, 2 × 30, 2 × 60, and 2 × 150 s post-injection of ^15^O-water). After a bolus injection of ~370 MBq of ^18^F-FDDNP, dynamic PET scans were acquired for 65 min, with a scanning protocol of 6 × 30, 4 × 180, and 10 × 300 s. No blood sample was obtained for ^18^F-FDDNP PET studies. Raw PET data were reconstructed with CAPP software (Siemens/CTI) on SUN workstations (Sun Microsystems) using a filtered backprojection algorithm (Hann filter cutoff at 0.3 of the Nyquist frequency) with correction for randoms, dead-time, scatter, detector normalization, photon attenuation, and radioactive decay.

### Image analysis

#### Parametric maps of physiological parameters

Quantitative parametric map of CBF was generated by voxel-wise fitting the one-tissue, three-parameter model to the measured ^15^O-water kinetics in brain tissue described by the following equations [26, 27]:

1 $$ \frac{{{\text{d}}C_{\text{T}} (t)}}{{{\text{d}}t}} = K_{1} C_{\text{b}} (t) - k_{2} C_{\text{T}} (t) $$

2 $$ C_{\text{ROI}} (t) = C_{\text{T}} (t) + V_{\text{b}} C_{\text{b}} (t), $$

where *C*_T_(*t*) is the activity concentration of ^15^O-water in brain tissue, *C*_b_(*t*) is the activity concentration of ^15^O-water in arterial blood, *C*_ROI_(*t*) is the total activity concentration of ^15^O-water in the tissue RO) measured by PET, *K*_1_ is the CBF, *k*_2_ is the clearance rate constant, and *V*_b_ is the vascular volume within the ROI. The first-pass extraction fraction of water was fixed at 0.85. The delay and dispersion of the arterial input function was corrected by minimizing the residual sum-of-squared errors of model fitting to the whole-brain time–activity curve. Noise in the parametric CBF image was reduced using a linear ridge regression with spatial constraint [30].

Simplified reference-tissue model (SRTM) [31] has been shown to provide reliable fits to ^18^F-FDDNP kinetics in human brain [28]. This approach assumes that the rates of exchange between free and non-specific compartments are rapid so that they are kinetically indistinguishable; both reference and target regions have the same non-displaceable volume of distribution, and the reference region is devoid of specific/displaceable binding and can be described by a single compartment. Target tissue time course can be fitted to the SRTM using non-linear regression [31]:

3 $$ C_{\text{T}} (t) = R_{\text{I}} C_{\text{R}} (t) + \left( {k_{2} - \frac{{R_{\text{I}} k_{2} }}{1 + BP}} \right)C_{\text{R}} (t) \otimes e^{{ - \left( {k_{2} /\left( {1 + BP} \right)} \right)t}}, $$

where *C*_T_(*t*) is the time course of activity concentration in the target region, *C*_R_(*t*) is the time course of activity concentration in the reference region, *R*_I_ is the ratio of the tracer delivery in the target region compared to that in the reference region (i.e., relative perfusion between the target and reference regions), *k*_2_ is the efflux rate constant from the target region, *BP* is the binding potential, and ⊗ denotes the convolution integral operator. A basis function method [32] has been proposed for voxel-wise estimation of *R*_I_, *BP*, and *k*_2_ by rewriting Eq. (3) as

4 $$ C_{\text{T}} (t) = \alpha_{1} C_{\text{R}} (t) + \alpha_{2} B_{i} (t), $$

where *α*_1_ = *R*_I_, *α*_2_ = *k*_2_ − *R*_I_ *k*_2_/(1 + *BP*), *θ*_*i*_ = *k*_2_/(1 + *BP*), and $B_{i} (t) = C_{\text{R}} (t) \otimes e^{{ - \theta_{i} t}} .$ It can be seen that Eq. (4) can be solved using weighted linear least-squares by choosing *N* discrete values for *θ*_*i*_ that determine the basis functions *B*_*i*_(*t*). From the *N* sets of solution, the one with the lowest weighted residual sum-of-squared errors is chosen. For ^18^F-FDDNP, we found 100 discrete values for *θ*_*i*_ distributed logarithmically between 0.00636 and 1 min^−1^ to be sufficient. The cerebellar gray matter (CGM) was chosen as the reference region as β-amyloid plaques and neurofibrillary tangles have been demonstrated to be very low [33]. The CGM region was delineated based on an anatomically labeled atlas defined on a standardized brain template (to be described later).

#### Multimodality image registration

The ^15^O-water and ^18^F-FDDNP PET data were integrated over 0–10-min and 0–6-min post-injection, respectively, so as to enhance detection of distribution boundaries and cortical regions and to provide sufficient counts for accurate co-registration with MR image. To derive spatial mappings between structural (MR) and functional (PET) imaging data, the integrated ^15^O-water and ^18^F-FDDNP PET image data were separately co-registered to MR images using a 6-parameter rigid-body transformation and maximization of mutual information [34].

#### Symmetric diffeomorphic normalization

SyN uses diffeomorphisms as the transformation model to transform an image *S* (“source” image) to an image *T* (“target” or “template” image), both defined on an image domain Ω. A diffeomorphism $\phi$ of domain Ω is a one-to-one, differentiable, and invertible map with a differentiable inverse [35]. Define a spatial coordinate, **x**, a time variable, $t \in [ 0 , { 1}],$ a diffeomorphic space with homogeneous boundary conditions, Ψ, and a smooth velocity field at time *t*, **v**(**x**,*t*) on Ω, which is a square-integrable vector field, a family of diffeomorphic maps $\phi ({\mathbf{x}},t) \in \Uppsi$ along a geodesic connecting *S* and *T* can be constructed by integrating the time-dependent velocity fields governed by the following ordinary differential equation [35]:

5 $$ \frac{{{\text{d}}\phi ({\mathbf{x}},t)}}{{{\text{d}}t}} = {\mathbf{v}}\left( {\phi ({\mathbf{x}},t),t} \right) $$

with $\phi \left( {{\mathbf{x}},0} \right) = {\mathbf{x}}$ such that for a small change in *t* there is a small change in the diffeomorphism and for each *t* there is a unique diffeomorphism. The distance metric for the geodesic between $\phi \left( {{\mathbf{x}},0} \right)$ and $\phi \left( {{\mathbf{x}},1} \right),\; D_{\Uppsi } \left( {\phi \left( {{\mathbf{x}},0} \right),\phi \left( {{\mathbf{x}},1} \right)} \right),$ is defined by taking the infimum over all such paths in the diffeomorphic space [17]:

6 $$ D_{\Uppsi } \left( {\phi \left( {{\mathbf{x}},0} \right),\phi \left( {{\mathbf{x}},1} \right)} \right) = \mathop {\inf }\limits_{\phi } \int_{0}^{1} {\left\| {{\mathbf{v}}\left( {{\mathbf{x}},t} \right)} \right\|_{L} dt} $$

in which the functional norm $\left\| \cdot \right\|_{L}$ regularizes the velocity field via a linear differential operator *L* in the form of $L = a\nabla^{2} + b{\mathbf{I}},$ where *a* and *b* are constants, and **I** represents the identity. The geodesic distance between $\phi \left( {{\mathbf{x}},0} \right)$ and $\phi \left( {{\mathbf{x}},1} \right)$ is symmetric, i.e., $D_{\Uppsi } \left( {\phi \left( {{\mathbf{x}},0} \right),\phi \left( {{\mathbf{x}},1} \right)} \right) = D_{\Uppsi } \left( {\phi \left( {{\mathbf{x}},1} \right),\phi \left( {{\mathbf{x}},0} \right)} \right).$ The diffeomorphisms also allow $\phi$ to be decomposed into two transformation mappings $\phi_{1} \left( {{\mathbf{x}},t} \right)$ and $\phi_{2} \left( {{\mathbf{x}},t} \right)$ traversing in opposite direction in time. Those transformations are composed in such a way that *S* and *T* contribute equally to the geodesic, and thereby symmetrizing the warping between *S* and *T* so that the same deformation is computed, regardless of the chosen similarity metric and the directionality of image warping [21, 22].

Assume that **x** and **z** are spatial coordinates that represent the same position of some anatomic structure in images *S* and *T*, respectively, we have, for all $t \in [ 0 , { 1}],\;\phi_{1} \left( {{\mathbf{x}},1} \right) = \phi_{2}^{ - 1} \left( {\phi_{1} \left( {{\mathbf{x}},t} \right),1 - t} \right) = {\mathbf{z}}$ and $\phi_{2} \left( {{\mathbf{z}},1 - t} \right) = \phi_{2} \left( {\phi_{1} \left( {{\mathbf{x}},1} \right),1 - t} \right) = \phi_{1} \left( {{\mathbf{x}},t} \right)$ for intermediate points along the geodesic parametrized with respect to both endpoints. Define $S^{\ast} = S\left( {\phi_{1} \left( {{\mathbf{x}},\bar{t}} \right)} \right),$ $T^{\ast} = T\left( {\phi_{2} \left( {{\mathbf{x}},\bar{t}} \right)} \right),$ and their local mean-subtracted images as $\bar{S}\left( {\mathbf{x}} \right) = S^{\ast} \left( {\mathbf{x}} \right) - \mu_{{S^{\ast} }} \left( {\mathbf{x}} \right)$, $\bar{T}\left( {\mathbf{x}} \right) = T^{\ast} \left( {\mathbf{x}} \right) - \mu_{{T^{\ast} }} \left( {\mathbf{x}} \right),$ where $\mu_{{S^{\ast} }}$ and $\mu_{{T^{\ast} }}$ are computed over a local *n* ^d^ window (i.e., a radius of *n* voxels and *d* is the image dimension) centered at each voxel position *x*, the following variational energy function generalized from inexact image matching [15, 17, 18] can be derived for optimization in diffeomorphic SyN [22]:

7 $$ E\left( {S,T} \right) = \mathop {\inf }\limits_{{\phi_{1} }} \mathop {\inf }\limits_{{\phi_{2} }} \int_{0}^{{\bar{t}}} {\left[ {\left\| {{\mathbf{v}}_{1} \left( {{\mathbf{x}},t} \right)} \right\|_{L}^{2}\,+\, \left\| {{\mathbf{v}}_{2} \left( {{\mathbf{x}},t} \right)} \right\|_{L}^{2} } \right]{\text{d}}t} + \int_{\Upomega } {\rho \left( {\bar{S},\bar{T},{\mathbf{x}}} \right){\text{d}}\Upomega } $$

subject to $\bar{t} = 0.5$ and each $\phi_{i} \in \Uppsi$ the solution of ${{d\phi_{i} \left( {{\mathbf{x}},t} \right)} \mathord{\left/ {\vphantom {{d\phi_{i} \left( {{\mathbf{x}},t} \right)} {dt}}} \right. \kern-0pt} {dt}} = {\mathbf{v}}_{i} \left( {\phi_{i} \left( {{\mathbf{x}},t} \right),t} \right)$ with $\phi_{i} \left( {{\mathbf{x}},0} \right) = {\mathbf{I}},\;\phi_{i}^{ - 1} \left( {\phi_{i} } \right) = {\mathbf{I}}$ and $\phi_{i} \left( {\phi_{i}^{ - 1} } \right) = {\mathbf{I}}.$ The first term on the right side of Eq. (7) gives the squared distance metric for the geodesic between $\phi \left( {{\mathbf{x}},0} \right)$ and $\phi \left( {{\mathbf{x}},1} \right)$ equivalent to that defined by Eq. (6) but it is computed through $\phi_{1}$ and $\phi_{2}$ instead, whereas the second term gives the similarity metric between $\bar{S}$ and $\bar{T}.$ While several different similarity metrics can be used with diffeomorphic SyN, localized cross-correlation was selected in this study as it depends only on estimates of the local image mean and variance and has shown to perform well in brain image registration [22, 24, 36]. The localized (squared) cross-correlation can be calculated as

8 $$ \rho \left( {\bar{S},\bar{T},{\mathbf{x}}} \right) = \frac{{\left\langle {\bar{S},\bar{T}} \right\rangle^{2} }}{{\left\langle {\bar{S},\bar{S}} \right\rangle \left\langle {\bar{T},\bar{T}} \right\rangle }}, $$

where $\left\langle { \cdot , \cdot } \right\rangle$ denotes the inner product operation over a local *n* ^d^ correlation window centered at each voxel position *x*. Optimizing Eq. (7) with respect to $\phi_{1}$ and $\phi_{2}$ at $\bar{t} = 0.5$ yields a set of Euler–Lagrange equations, the solutions of which are computed iteratively at multiple levels of resolution until the maximum number of iterations is reached or the similarity metric could not be further improved [22, 23]. Upon convergence, the SyN transformation from *S* to *T* is calculated as $\phi_{1} \left( {{\mathbf{x}},1} \right) = \phi_{2}^{ - 1} \left( {\phi_{1} \left( {{\mathbf{x}},0.5} \right),0.5} \right)$ and the inverse is given by $\phi_{2} \left( {{\mathbf{z}},1} \right) = \phi_{1}^{ - 1} \left( {\phi_{2} \left( {{\mathbf{z}},0.5} \right),0.5} \right).$

#### Constrained cost-function masking

In the presence of a focal lesion, standard warping with SyN may seem inappropriate as the assumption of a one-to-one mapping between the source and the template images is violated because of the abnormal shapes and intensity values of the focal lesion that cause a mismatch between both images and bias the cost function being optimized substantially. Currently, the cost-function masking (CFM) technique [37] is widely used to overcome difficulties encountered when normalizing brains with focal lesions. The main idea of CFM is to remove the contribution of focal lesions to the cost function by zeroing out all voxels within lesions. However, this approach is limited when the patients have large or bilateral lesions [38], which are not uncommon in TBI patients. In this study, we used SyN in conjunction with a constrained cost-function masking (CCFM) approach (SyN-CCFM) [23, 39] for handling brain warping in the presence of focal abnormality as a missing-data problem. It takes advantage of the fact that diffeomorphic mappings are determined by the velocity field which is spatially smooth. Thus, the unknown velocity field parameters within the lesion can be estimated and inferred from the velocity field parameters near and exterior to the lesion boundaries. In this way, the lesioned areas are constrained to be smoothly deformed in the most probable way that follows the deformation of the surrounding intact brain tissues, which may have gone through a large degree of transformation during the spatial normalization process.

#### Brain template and anatomical labeling

A high-resolution (1 × 1 × 1 mm^3^ voxels) single-subject T1-weighted MR brain template [40] provided by Montreal Neurological Institute (MNI) was chosen as the common stereotaxic space for matching all structural and functional imaging data to facilitate comparisons across subjects, mapping of 3D ROIs between different spaces, and anatomical labeling. Cortical and subcortical gray matter ROIs from the well-validated automated anatomical labeling (AAL) atlas [10] defined on the same MR brain template were used to examine regional physiological parameter values from the functional PET imaging data. Bilateral 3D ROIs were also manually drawn over the centrum semiovale using the ITK-SNAP software (http://www.itksnap.org/) for subcortical white matter region (SWM), which is not available in the AAL atlas.

### Data analysis

**Fig. 1:**
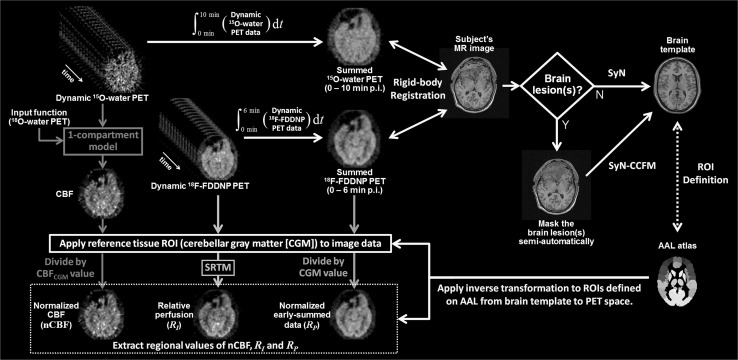
The complete schematics for the proposed workflow solution. Summed PET data were used for rigid-body co-registration with the structural MR image, which was spatially normalized to a standard brain template in the MNI space using SyN (and SyN-CCFM in the presence of focal lesion). Once the spatial correspondence was established, a set of ROIs taken from the AAL atlas defined in the MNI space was mapped back to the PET space to extract the cerebellar gray matter (for ^18^F-FDDNP). Parametric images of ^15^O-water PET (CBF and nCBF, 0–10 min) and ^18^F-FDDNP PET [*R*_I_ and *R*_P_ (0–6 min)] data were constructed using the corresponding methods (see Sect. 2). The same set of ROIs was applied to the CBF and relative-perfusion (*R*_*I*_ and *R*_*P*_) images to derive the associated regional values using the combined transformation between template and PET space

The complete workflow solution was implemented using MATLAB (The MathWorks, Natick, MA) and an overview of it is depicted in Fig. 1. Quantitative analyses of ^15^O-water and ^18^F-FDDNP PET imaging data were performed with programs developed and validated in-house. The ^15^O-water and ^18^F-FDDNP PET data were integrated over 0–10-min and 0–6-min post-injection, respectively, and were co-registered to the subject’s MR images using a rigid-body transformation as described before. Focal brain lesions, if present on the individual’s MR image, were masked by a semi-automatic active contour algorithm [41] implemented in the ITK-SNAP software (http://www.itksnap.org/), and the resulting masks were used in SyN-CCFM for warping the MNI single-subject brain template to the subject’s MR image. In the absence of brain lesion, regular SyN warping was used for matching between the MNI single-subject brain template and the subject’s MR image. Both rigid-body co-registration and diffeomorphic normalization were performed using ANTs [23] and the procedures were fully automated with a set of parameters (e.g., number of iterations and number of bins for histogram calculation, etc.) defined a priori or given by the user. For spatial normalization using SyN or SyN-CCFM, we chose to use four levels of resolution (from coarse to fine) with scaling factors of 8, 4, 2, and 1, with the maximum number of iterations set to 250 for all resolution levels. The CGM region was used as the reference-tissue for ^18^F-FDDNP PET and was taken from the gray matter areas defined over the cerebellum on the AAL atlas. It was then transferred using the concatenated transformation from MNI template space to PET space and projected onto the dynamic ^18^F-FDDNP PET images at all time frames to derive the volume-averaged reference-tissue TAC. Parametric images of CBF and *R*_*I*_ were constructed using the arterial input function of ^15^O-water and the reference-tissue (CGM) TAC, respectively. To compare with $R_{I}$ derived from ^18^F-FDDNP PET using SRTM, parametric CBF and early-summed ^18^F-FDDNP PET (0–6 min) images were divided by their values in CGM to create normalized CBF (nCBF) and normalized summed ^18^F-FDDNP ($R_{P}$) images, respectively. Regional physiological parameters (CBF, nCBF, $R_{I}$, and $R_{P}$) were extracted from 14 cortical, subcortical, and white matter ROIs defined on the AAL atlas using the combined transformations between template and PET space. Descriptive statistical results are presented as mean ± standard deviation (SD).

## Results

**Fig. 2:**
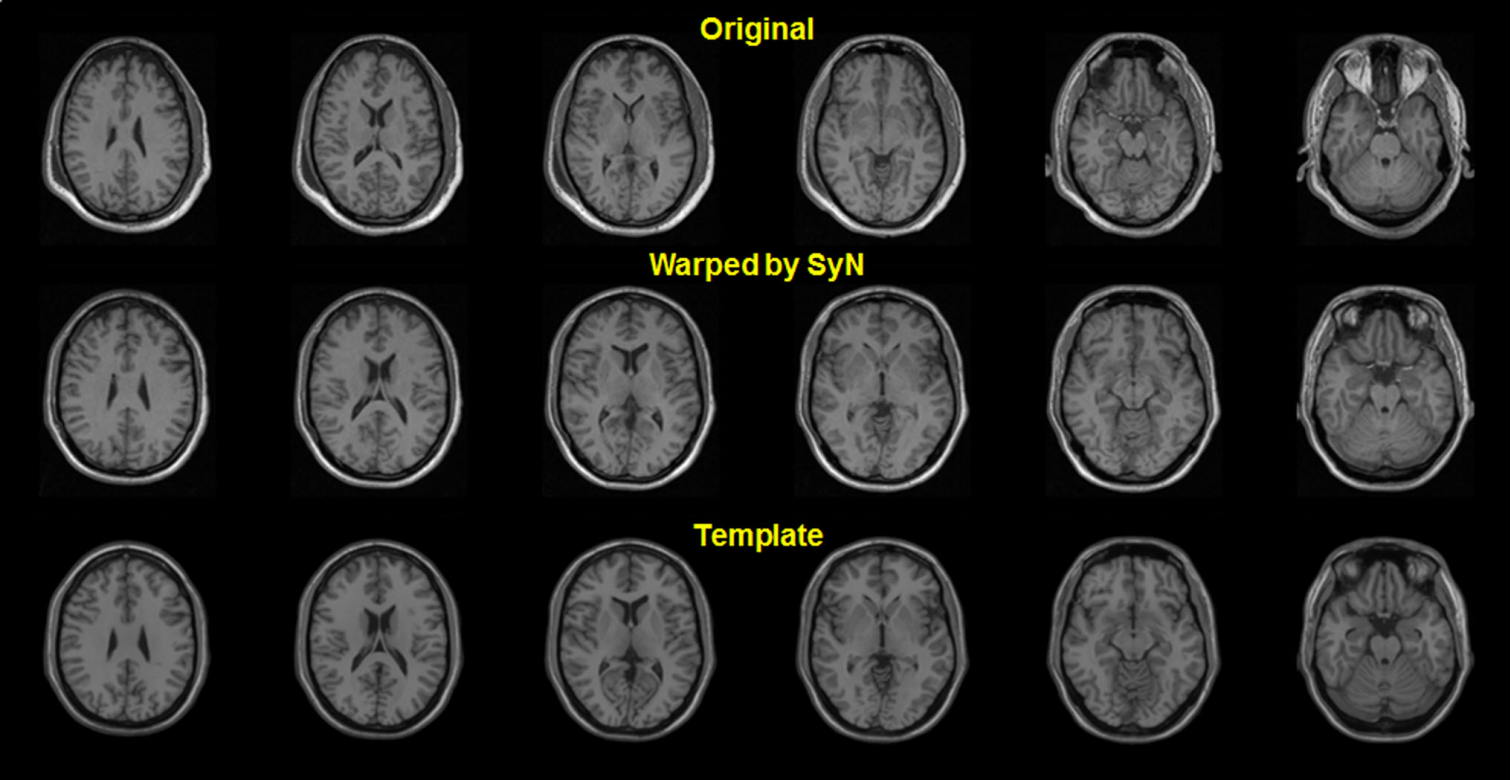
Comparison of the original image (*top row*), template (*bottom row*), and the warped original image to the template (*middle row*). Images are displayed in radiological convention. No focal lesion was observed in this subject (#2) based on the MR image

Figure 2 shows the SyN warping results of the brain of a TBI subject (#2), who had a right frontotemporoparietal craniotomy with evacuation of subdural hematoma on the day of injury. No obvious focal lesion was seen on the brain from the MR image. SyN was thus performed without using CCFM and the warping of the original brain to the template brain was almost perfect, as the ventricular space and much of the cortical and subcortical regions were well aligned, with the exception of the occipital lobe, where the shape and appearance of gyri were very difficult to capture because of their highly idiosyncrasy in that area. Moreover, SyN was able to provide a decent matching of the subject’s whole-head to the template despite the differences in shape and thickness of the skull.

**Fig. 3:**
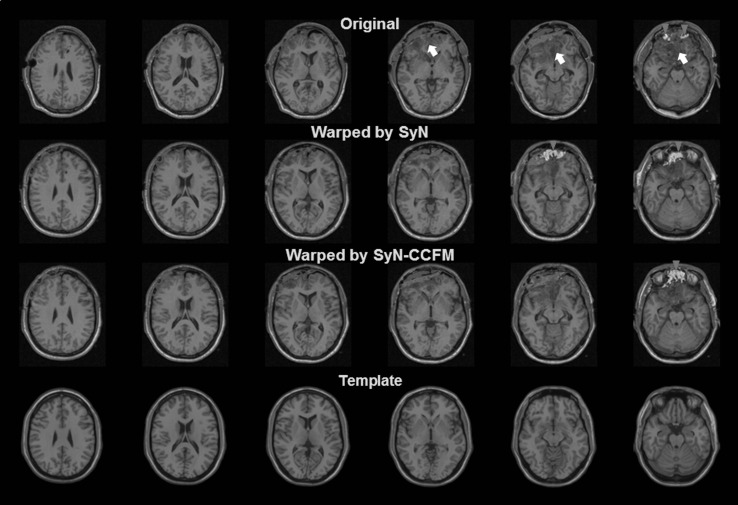
Comparison of warping between the original image (*top row*) of subject #1 and the template (*bottom row*). Shown also are the warped original image to the template with SyN only (*second row*) and with SyN-CCFM (*third row*). Images are displayed in radiological convention. Lesions that require masking are indicated by *white arrow* and *pink arrowhead*. Region with higher intensity values near the eyeballs (*pink arrowhead*) was pushed to the frontal area when SyN-CCFM was not used, but was well contained by the use of SyN-CCFM. (Color figure online)

**Fig. 4:**
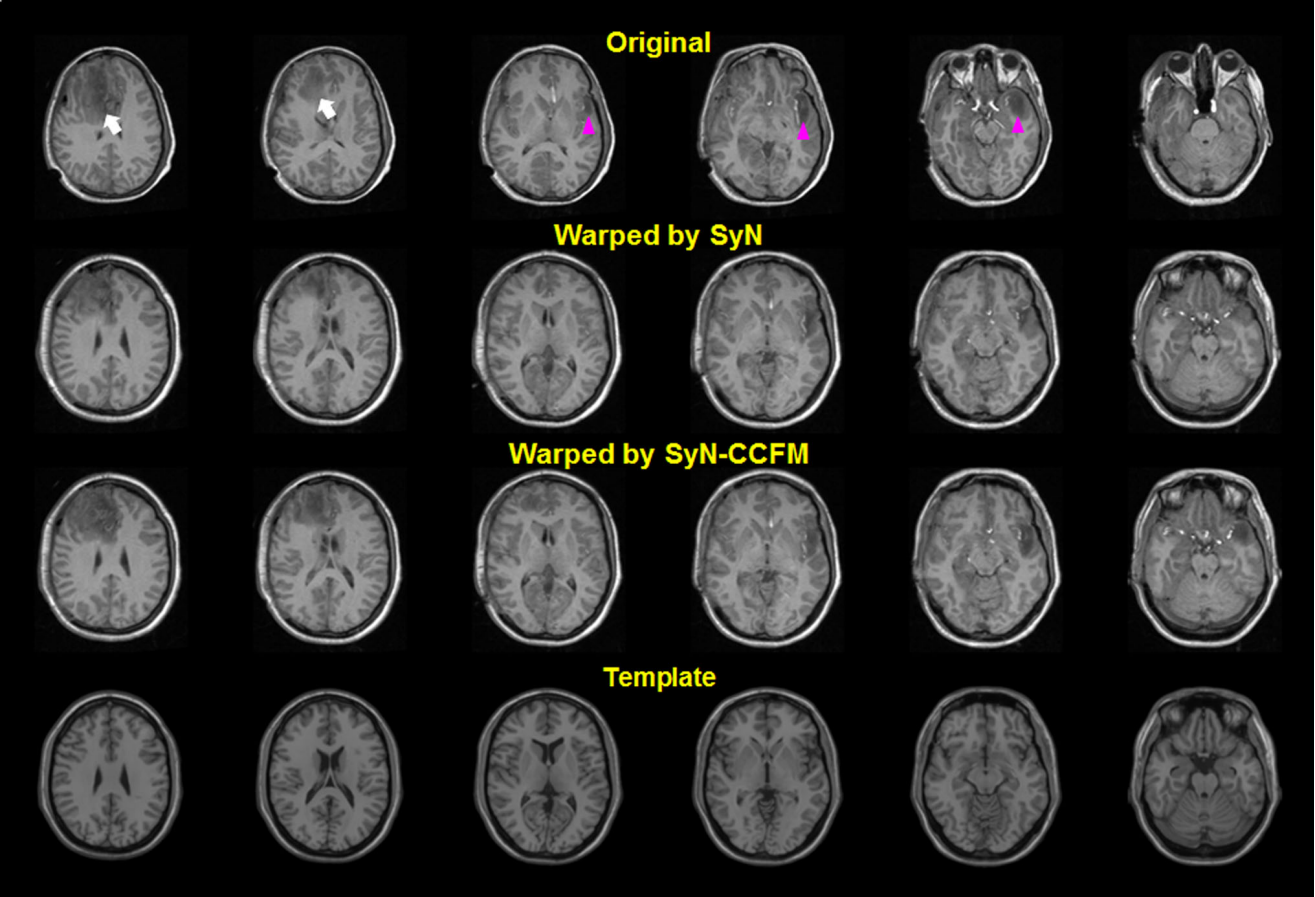
Comparison of warping between the original image (*top row*) of subject #3 and the template (*bottom row*). Shown also are the warped original image to the template with SyN only (*second row*) and with SyN-CCFM (*third row*). Images are displayed in radiological convention. A large lesion (*white arrow*) was found on the right frontal lobe and extended to the left frontal lobe along with a shrunken lateral ventricle. A smaller lesion was also seen on the left lateral temporal area (*pink arrowhead*). (Color figure online)

Figure 3 illustrates the use of SyN and SyN-CCFM in normalizing the brain of a TBI subject (#1), who had undergone surgical procedures for evacuation of bilateral frontal epidural and subdural hematomas as well as intraparenchymal hematoma on the day of injury. Focal lesions were observed in the frontal region and near the eyeballs based on the MR image. With SyN alone, the region with atypically high voxel intensity was “pushed” and extended to the orbital gyrus and the more superior portion of the frontal region. This is likely because of the disproportionately high intensity for voxels within the focal lesion which causes the optimization algorithm to attempt further reduction of the cost function by minimizing the mismatch between the original and the template images at the site of the lesion, even though other areas have already been aligned well. In contrast, SyN-CCFM gave reasonable warped results due to the use of a lesion mask, the voxels within which were treated as missing-data by the optimization algorithm and the deformation field within the mask was estimated and inferred from that given by the surrounding tissues. The overall shape and appearance of the brain, gyri, and ventricular space are well matched to that of the template brain. Figure 4 illustrates another case of comparison between SyN with and without CCFM for brain warping of subject #3 who had a large lesion that occupied a significant portion of right frontal lobe and a moderate-sized lesion in the left lateral temporal area. Again, the warping results favor the use of SyN-CCFM for normalizing injured brain with focal gross pathology.

**Fig. 5:**
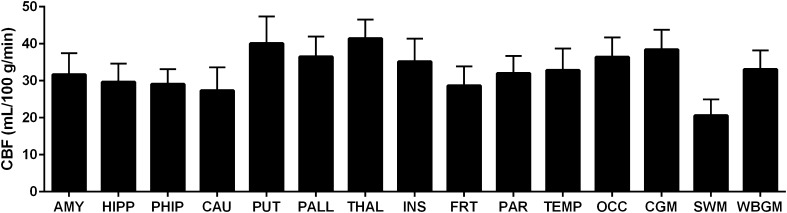
Mean regional CBF obtained using ^15^O-water PET (*n* = 6). *Error bars* represent 1 SD. *AMY* amygdala, *HIPP* hippocampus, *PHIP* parahippocampus, *CAU* caudate nucleus, *PUT* putamen, *PALL* pallidum, *THAL* thalamus, *INS* insula, *FRT* frontal, *PAR* parietal, *TEMP* temporal, *OCC* occipital, *CGM* cerebellar gray matter, *SWM* subcortical white matter (centrum semiovale), *WBGM* whole-brain-averaged gray matter

**Fig. 6:**
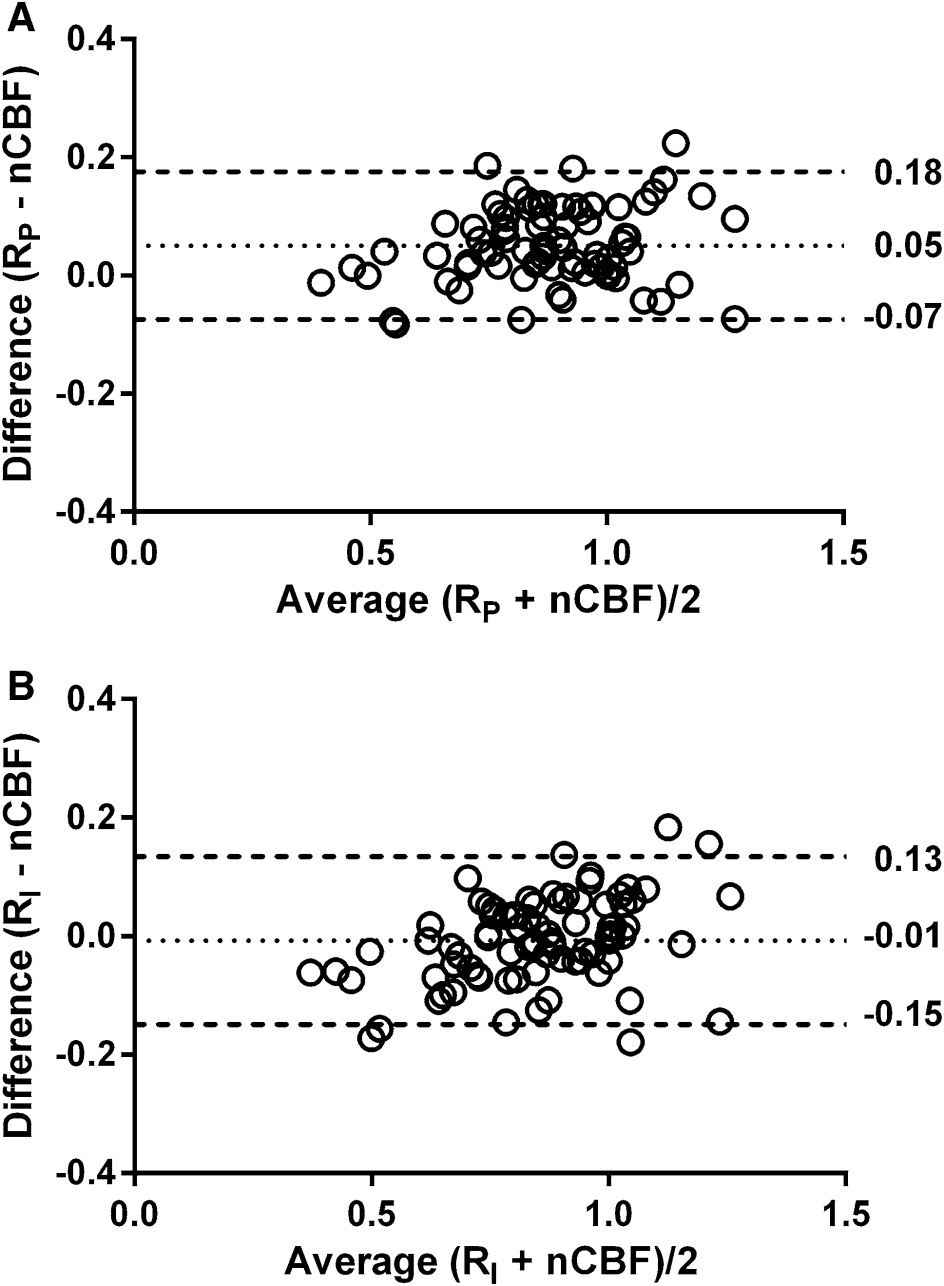
Bland–Altman plots of differences, showing the limits of agreement between nCBF and (**a**) *R*_P_ and (**b**) *R*_I_ over all regions and patients. The *dotted lines* represent the mean difference and the *dashed lines* represent the 95 % confidence interval for the difference (mean ± 1.96 SD)

Regional CBF and their variability are shown in Fig. 5. Because the patients were sedated during PET scanning, CBF was reduced globally. Mean CBF in whole-brain gray matter was 33.1 ± 5.1 mL/100 g/min and was calculated by averaging CBF in 13 cortical and subcortical gray matter ROIs extracted from the AAL atlas. Averaged CBF was 38.4 ± 5.4 mL/100 g/min in cerebellar gray matter and 20.5 ± 4.1 mL/100 g/min in SWM (centrum semiovale). Coefficient of variation in CBF was similar among different regions, ranging from 14 to 23 %, with a mean of 17 %. The whole-brain-averaged gray/white ratio was 1.65 ± 0.3 (*n* = 6). Figure 6 shows the Bland–Altman plots of difference, showing the limits of agreement between *R*_P_ and *R*_I_ versus nCBF over all regions and patients. As can be seen from the plots, majority of data points lie within the 95 % confidence interval for the difference (mean ± 1.96 SD), and the mean biases were close to zero, suggesting that there were good overall agreements between nCBF, *R*_P_ and *R*_I_.

## Discussion

The ultimate goal of the workflow solution is to establish spatial correspondences between imaging data obtained with different modalities and a high-resolution brain template chosen by the user with no (or minimal) human intervention throughout the processing of the imaging data, and subsequently facilitating anatomic labeling and group analysis. Central to the workflow solution is the capability to closely normalize an individual’s brain to a standard brain template defined in a common space while maintaining the integrity of brain structures. In general, spatial normalization seeks to estimate an optimal transformation map $\phi$ that brings an image *S* closest to an image *T* by minimizing a cost function that describes the similarity between the images under certain matching criteria. Ideally, the transformation mapping $\phi$ should be one-to-one correspondence, smooth, differentiable, and symmetric (i.e., independent of the directionality between *S* and *T*). The idea of inverse consistency was first put forward by Thirion [14] and was generalized by Christensen and Johnson [42] in their inverse consistent image registration (ICIR) method, where symmetry is approximated by including a variational penalty term in the optimization algorithm. However, the inverses for traversing between *S* and *T* are not guaranteed to exist as the optimization is not performed in diffeomorphic space. In contrast, SyN was formulated using diffeomorphism and guarantees that identical results are obtained regardless of the input order between *S* and *T* and that exact inverse transformations exist [21, 22].

While pre-processing the structural imaging data such as brain extraction (or skull-stripping) [43], tissue classification [44], and bias-field correction [45] is essential to facilitate accurate image analysis, it is important to recognize that fully automatic procedures could not be applied routinely without quality check by human experts, regardless of how sophisticated the pre-processing algorithm is. Different from spatial normalization of normal brains and atrophied brains caused by neurodegenerative disorders, patients with TBI typically presented with a combination of diffuse axonal injury and gross brain pathologies, and the injured locations vary among patients having different causes of injury and degrees of brain damage. Thus, brain extraction and tissue classification become a challenging task and require some level of user supervision to guide identification, localization, and isolation of abnormalities in the image. Although the general consensus of Klein et al. [24] suggested that image registration methods would perform better on properly skull-stripped images than on whole-head images, no study has yet been published that made this comparison. In this work, we did not apply skull-stripping to the individual MR image or the brain template as we observed that larger distortion and mismatch usually occur along the brain surface in the absence of skull in either set of image (data not shown). This is likely because of the missing “information” outside of the brain surface that would have been incorporated by the warping algorithm as parts of the similarity metric for matching and as boundary conditions imposed on the brain surface for constraining its deformation, if the non-brain regions were not removed. In line with recent findings [22, 24, 36], our results also show that the local cross-correlation, which depends only on local image mean and variance and can be calculated rapidly and accurately with relatively few samples, allows for robust matching between the brain template and the subject’s brain MR image with morphological brain changes or in the presence of intensity inhomogeneity caused by magnetic field imperfections that degrade both image quality and tissue classification accuracy, thereby obviating the need of bias-field correction and tissue classification as pre-processing steps required by other image normalization methods such as DARTEL [19] and FNIRT [20]. As such, the proposed workflow solution can be used in studies where only non-T1-weighted MR images are available.

It is important to note that rigid-body co-registration (between functional PET data and individual MR brain) and spatial normalization (between individual MR brains and the brain template) are independent processes. If the transformations from template to PET space (or vice versa) were performed in the most straightforward way by generating the intermediate data in the individual’s MR space, subtle errors could be introduced through reslicing and interpolation of image volumes with different resolutions. In this study, the forward and inverse transformations between template and PET spaces were composed by concatenating a series of transformations prior to transforming the image. In this way, interpolation error due to reslicing and resampling of image volume is minimized, whereas the storage space for saving intermediate data is not required. Composition of transformations by concatenation can easily be generalized and applied to cases where more steps of image co-registration and/or non-linear warping are added to the workflow. For example, if one had computed all the transformations to a given template but another template image was later added, one would have to perform the non-linear warping to the new template and discard the warping results to the original template. With the concatenation of transformations, one would need to establish the transformation between the original template and the newly added template image, thereby saving significant amount of time and effort. Given that SyN consistently ranked the best for all error measures tests and for all label sets [24], we expect that the results obtained indirectly by concatenating a series of transformations would only be marginally different from those obtained by a direct warp to the new template, although more work will need to be conducted to evaluate the error bounds between these approaches.

One of the major limitations of this work is the lack of anatomical-based evaluation with manual labeling of brain regions which serves as the reference standard for comparing with the results from automated anatomical labeling. However, manual labeling is tedious and time-prohibitive for analyzing even a modest number of studies, and is subject to intra- and inter-rater variability [2, 6, 7]. In contrast, normalizing brains to one another or to a standard brain template for reproducible determination of anatomical correspondence is almost performed universally [24] and is well accepted in the field of neuroimaging where many tools (e.g., SPM [11] and AIR [12]) have been developed for this purpose and for more sophisticated statistical analyses conducted at voxel and cluster levels. We have investigated the validity of the correspondence between the physiological parameter extracted from the regions defined on the common space and those defined on the subject space for brain datasets obtained from a cohort consisting of cognitively normal subjects and patients with dementia or mild cognitive impairment, having moderate to severe cortical degeneration [46]. Using the Dice overlap statistic (*κ*) [47], which measures spatial overlap between two regions defined in a different way and has a range of 0 (i.e., no spatial overlap) and 1 (i.e., complete overlap), our results showed that *κ* > 0.7 for small structures and *κ* > 0.9 for gray and white matter, thus indicating excellent agreement which is generally defined as *κ* > 0.7 [48]. In spite of the differences in quantitation methodologies and patient characteristics, the whole-brain averaged CBF derived in this study is in generally good agreement with those published previously [49, 50]. The main advance of this study is that CBF in various brain regions can be quantified using the proposed workflow solution with relative ease while removing some sources of experimental variability. A thorough comparative evaluation on the physiological issue and the biological significance for regional flow measurements obtained from ^15^O-water PET and ^18^F-FDDNP PET will be detailed in another report.

Most of the existing software packages (e.g., 3DSlicer and FreeSurfer) are primarily designed for processing and analyzing structural brain MR images. To our knowledge, no tool has yet been available for streamlining image registration, non-linear spatial normalization, voxel-wise kinetic analysis, and automated labeling and ROI analysis of both structural MR images and dynamic PET datasets from multi-tracer studies for TBI. Our workflow solution integrates various specialized techniques for structural MR data and dynamic PET image analysis. We showcase the workflow solution using ^15^O-water PET and ^18^F-FDDNP PET which are only cases in point in this study. A wide variety of PET data analysis techniques can easily be adopted in the workflow solution to deal with tracer studies using different radiolabeled compound and imaging protocol. Unlike many other software packages that focus on the user-friendliness and interactive graphical user interface, we opted to implement our workflow solution in a script-oriented program using MATLAB, which is cross-platform and has a rich set of functions for high-performance scientific computation. We also put emphasis on minimal user input (to minimize as much of the operator error as possible), applicability in a busy clinical/research environment (where high-throughput automation and pipeline processing of multiple studies are desirable), and scalability (to accommodate changes in imaging protocols such as including PET studies from the same or different sessions, or adding functional MR studies to the workflow). The design philosophy thus enables the workflow solution to be portable to multiple computer platforms without the need to worry about incompatibility and dependency of the graphical libraries associated with different computing systems, and gives investigators a large degree of freedom to choose and use their favorite data visualization software to display, view, and manipulate the intermediate and final image results. The capability of the proposed workflow solution to normalize brain images with focal lesions and large deformations also allows it to process and analyze brain images having similar characteristics seen in different neurological diseases such as stroke and brain tumors. However, lesion masking performed either by an investigator or by a computer-aided method is needed for analyzing those cases. How precise the lesion mask is defined has been shown not to affect the brain warping results, as the main purpose of masking is to remove the contribution to the cost function due to atypical voxel intensity enclosed by the mask [37]. The time required for lesion masking can thus be substantially reduced by using a semi-automated masking approach based on an active contour algorithm [41] as in this study. Fully automated algorithms would be of great use for lesion masking, but much research is still needed to improve tissue classification/segmentation accuracy.

## Conclusions

In this paper, we proposed a workflow solution framework that combined the use of non-linear brain warping of structural MR images and anatomical ROI labeling to automatically derive physiological parameters from functional imaging of patients having acute TBI. We presented how we combined various image processing and parametric imaging approaches for analyzing structural MR images and dynamic multi-tracer PET scans. The workflow solution was then applied to quantify regional CBF in TBI patients. The proposed framework offers improvement over existing manual ROI approach (which is time-consuming and subject to reproducibility errors) through automated anatomical labeling of a standard brain in a common stereotaxic space, and is expected to be useful to a wide variety of neuroimaging applications that requires aggregation and regionalization of imaging data obtained from multiple modalities as well as standardization and automation of image processing and analysis with minimal user intervention.
